# Do Infants in the First Year of Life Expect Equal Resource Allocations?

**DOI:** 10.3389/fpsyg.2019.00116

**Published:** 2019-02-19

**Authors:** Melody Buyukozer Dawkins, Stephanie Sloane, Renée Baillargeon

**Affiliations:** ^1^Department of Psychology, University of Illinois at Urbana-Champaign, IL, United States; ^2^Department of Human Development and Family Studies, University of Illinois at Urbana-Champaign, IL, United States

**Keywords:** infancy, social cognition, morality, fairness, equality, resource allocation, numerical cognition, first year

## Abstract

Recent research has provided converging evidence, using multiple tasks, of sensitivity to fairness in the second year of life. In contrast, findings in the first year have been mixed, leaving it unclear whether young infants possess an expectation of fairness. The present research examined the possibility that young infants might expect windfall resources to be divided equally between similar recipients, but might demonstrate this expectation only under very simple conditions. In three violation-of-expectation experiments, 9-month-olds (*N* = 120) expected an experimenter to divide two cookies equally between two animated puppets (1:1), and they detected a violation when she divided them unfairly instead (2:0). The same positive result was obtained whether the experimenter gave the cookies one by one to the puppets (Experiments 1–2) or first separated them onto placemats and then gave each puppet a placemat (Experiment 3). However, a negative result was obtained when four (as opposed to two) cookies were allocated: Infants looked about equally whether they saw a fair (2:2) or an unfair (3:1) distribution (Experiment 3). A final experiment revealed that 4-month-olds (*N* = 40) also expected an experimenter to distribute two cookies equally between two animated puppets (Experiment 4). Together, these and various control results support two broad conclusions. First, sensitivity to fairness emerges very early in life, consistent with claims that an abstract expectation of fairness is part of the basic structure of human moral cognition. Second, this expectation can at first be observed only under simple conditions, and speculations are offered as to why this might be the case.

## Introduction

Over the past decade, developmental researchers have begun to systematically explore the foundations of moral cognition in infancy (e.g., Hamlin, 2013b; Spelke et al., 2013; Thomsen and Carey, 2013; Tomasello and Vaish, 2013; Paulus, 2014; Baillargeon et al., 2015; Martin and Olson, 2015; Bloom and Wynn, 2016; Davidov et al., 2016; Warneken, 2016; Liberman et al., 2017; Sommerville and Enright, 2018). In particular, several investigations have sought to uncover the early precursors of adults’ and older children’s well-established concern for fairness (e.g., Dawes et al., 2007; Fehr et al., 2008; Olson and Spelke, 2008; Rochat et al., 2009; Ng et al., 2011; Baumard et al., 2012; Shaw and Olson, 2012; Smith et al., 2013; Hamann et al., 2014; McAuliffe et al., 2015, 2017; Renno and Shutts, 2015). In this report, we focus on infants’ sensitivity to fairness in third-party situations where windfall resources are divided, either fairly or unfairly, between two similar recipients. In the next sections, we first summarize prior findings from relevant tasks. As will become clear, positive results have been obtained in the second year of life with a variety of tasks, providing converging evidence of sensitivity to fairness in older infants. In contrast, results in the first year have been mixed, leaving it unclear whether young infants possess an expectation of fairness. Next, we introduce the present experiments, which sought to reconcile the divergent findings that have been obtained with young infants and, in so doing, to ascertain at what age and under what conditions sensitivity to fairness can be observed in the first year of life.

We reasoned that such evidence would be important for at least two reasons: It would constrain theoretical accounts of the mechanisms by which an expectation of fairness first emerges in infancy, and it would help identify some of the factors that affect under what conditions this expectation is likely to be demonstrated.

### Findings With Older Infants

Evidence of sensitivity to fairness in the second year of life comes from at least three different tasks. In *allocation-outcome* tasks, a distributor divides resources either equally (*equal* event) or unequally (*unequal* event) between two similar recipients. The rationale is that if infants expect the distributor to act fairly, then they should look longer when this expectation is violated in the unequal event. To date, positive results have been obtained in four published reports with infants ages 15–19 months (Schmidt and Sommerville, 2011; Sloane et al., 2012; Enright et al., 2017; Bian et al., 2018; see also Tatone and Csibra, 2018). These reports varied along multiple dimensions, including whether the events were videotaped or live; whether the distributor and recipients were humans or puppets; whether infants saw a single distribution event followed by still-frame images depicting the equal and unequal outcomes or separate distribution events for the two outcomes; and whether the allocated resources comprised four items, with 2:2 and 3:1 outcomes (Schmidt and Sommerville, 2011; Enright et al., 2017), or two items, with 1:1 and 2:0 outcomes (Sloane et al., 2012; Bian et al., 2018). Positive results have also been obtained with infants ages 12–15 months under limited conditions (Ziv and Sommerville, 2017): When shown a videotaped event in which four items were distributed, followed by simultaneous still-frame images depicting equal (2:2) and unequal (3:1) outcomes, infants with one or more older siblings looked significantly longer at the unequal outcome. In contrast, infants without siblings tended to look equally at the two outcomes, as did 12-month-olds who were shown the two outcomes successively, rather than simultaneously (Sommerville et al., 2013; Tatone and Csibra, 2018).

In *affiliative-preference* tasks, one distributor divides resources equally between two recipients (*fair-distributor* event), and another distributor divides resources unequally between the same recipients *(unfair-distributor* event). Next, infants are encouraged to choose between the two distributors or to select one of two identical toys offered by the distributors. The rationale is that if infants expect a fair distribution, then they may prefer the fair over the unfair distributor, just as they prefer individuals who produce helpful as opposed to harmful actions (e.g., Hamlin et al., 2007, 2013a; Hamlin and Wynn, 2011). To date, positive results have been obtained with 16-month-olds using 2:0 violations (Geraci and Surian, 2011), with 15-month-olds using 3:1 violations (Burns and Sommerville, 2014), and with 13- and 17-month-olds using 5:1 violations (Lucca et al., 2018). In each report, infants were significantly more likely to prefer or endorse the fair over the unfair distributor.

In *reward*/*punishment* tasks, infants first see a fair and an unfair distributor divide resources between two recipients, and then the distributors are rewarded or punished for their actions. In one experiment (DesChamps et al., 2016), for example, 15-month-olds first saw videotaped events in which two women distributed four or six items; one woman did so fairly, and the other did so unfairly, resulting in 3:1 or 5:1 violations. Next, photos of the two women were presented simultaneously, accompanied by a series of seven statements spoken by a disembodied voice. In the *reward* condition, the statements conveyed praise (e.g., “She’s a good girl!”); in the *punishment* condition, they conveyed admonishment (e.g., “She’s a bad girl!”). Infants looked significantly longer at the unfair distributor in the reward condition, but looked equally at the two distributors in the punishment condition. One possible interpretation of these findings is that two separate tendencies contributed to infants’ responses: a tendency to look longer at the distributor who did not match the statements spoken, and a tendency to look longer at the unfair distributor (perhaps due to a vigilance or negativity bias; e.g., Kinzler and Shutts, 2008; Vaish et al., 2008; Baltazar et al., 2012). In the reward condition, these two tendencies combined, leading infants to look longer at the unfair distributor; in the punishment condition, these two tendencies canceled each other, resulting in equal looking times at the two distributors.

### Findings With Younger Infants

Sensitivity to fairness in the first year of life has been examined using the same types of tasks as with older infants. Reports using allocation-outcome tasks have yielded mixed results. When tested with computer-animated events showing a two-item distribution, 10-month-olds looked significantly longer at the unequal (2:0) than at the equal (1:1) event (Meristo et al., 2016). However, when tested with videotaped events showing a four-item distribution, with the final still-frame images depicting the unequal (3:1) and equal (2:2) outcomes presented simultaneously, 9- and 6-month-olds tended to look equally at the two outcomes, and this was true whether or not they had older siblings (Ziv and Sommerville, 2017).

A report using an affiliative-preference task also yielded negative results. After watching computer-animated events in which a fair and an unfair distributor divided two items between two recipients, 16-month-olds significantly preferred the fair over the unfair distributor (“Which one do you want? Pick it up!”), but 10-month-olds chose randomly between them (Geraci and Surian, 2011).

Finally, reports using reward/punishment tasks have yielded inconsistent results. In one report (Meristo and Surian, 2013), 10-month-olds first saw computer-animated events in which a fair and an unfair distributor divided two items between two recipients; a bystander either observed these distributions (*informed* condition) or was prevented from doing so by a partial barrier (*uninformed* condition). Next, the bystander gave a reward (a strawberry) to either the fair or the unfair distributor.^1^ Infants in the informed condition looked significantly longer when the bystander rewarded the unfair as opposed to the fair distributor, whereas infants in the uninformed condition looked equally at the two events, suggesting that they understood that the bystander lacked the necessary information to distinguish between the distributors. However, in additional experiments (Meristo and Surian, 2013, 2014), 10-month-olds also looked significantly longer when a newcomer who was absent during the distributors’ actions, and therefore should have been uninformed, (a) rewarded the unfair as opposed to the fair distributor or (b) punished the unfair as opposed to the fair distributor (e.g., by taking away a strawberry).

### Two Hypotheses

The results reviewed in the preceding sections indicate that by the second year of life, infants expect a distributor to divide resources fairly between two similar recipients: They detect a violation when shown unequal distributions, they prefer fair over unfair distributors, and they selectively associate praise with fair distributors and admonishment with unfair distributors. In contrast, findings with infants in the first year of life were mixed, leaving it unclear at what age and under what conditions young infants first demonstrate an expectation of fairness. In particular, consider the divergent results from the allocation-outcome tasks of Ziv and Sommerville (2017) and Meristo et al. (2016). At least two hypotheses can be offered for these conflicting results; these hypotheses focus on different procedural variations between the two tasks and invoke different mechanisms to explain the emergence of fairness in infancy.

One (*shift*) hypothesis focuses on the different ages tested in the two tasks: Ziv and Sommerville (2017) obtained negative results with 6- and 9-month-olds, while Meristo et al. (2016) obtained positive results with 10-month-olds. According to this hypothesis, an important developmental shift takes place at about 10 months of age that leads to the acquisition of expectations about fairness. This shift occurs largely through socialization processes: As infants interact with others (e.g., parents, other caregivers, siblings) in their everyday social environments, they come to learn that resources are typically distributed equally between similar recipients (e.g., Sommerville et al., 2013; Bloom and Wynn, 2016; Ziv and Sommerville, 2017). From this perspective, it would make sense that even at 12–15 months of age, infants with older siblings were more likely to demonstrate sensitivity to fairness than were infants without siblings (Ziv and Sommerville, 2017). The presence of older siblings would result in more opportunities to learn about fairness and hence would “spur the developmental shift in infants’ fairness expectations” (Ziv and Sommerville, 2017, p. 1044).

The other (*continuity*) hypothesis focuses on the different fairness violations used in the two tasks: Ziv and Sommerville (2017) obtained negative results with a 3:1 violation, while Meristo et al. (2016) obtained positive results with a 2:0 violation. According to this explanation, an abstract expectation of fairness emerges very early in life, as part of the basic structure of human moral cognition (e.g., Shweder et al., 1997; Dawes et al., 2007; Jackendoff, 2007; Premack, 2007; Rai and Fiske, 2011; Baumard et al., 2013; Graham et al., 2013; Baillargeon et al., 2015; Bian et al., 2018; Buyukozer Dawkins et al., in press). However, this expectation can at first be demonstrated only under limited conditions, which gradually broaden with experience. For example, it might be that young infants are initially able to process distributions of two items, but not distributions of four or more items; that they are initially able to detect qualitative violations, in which one recipient gets something and the other gets nothing (e.g., a 2:0 or a 4:0 violation), but not quantitative violations, in which both recipients get something but in differing amounts (e.g., a 3:1 or a 7:1 violation); or that they are initially able to detect quantitative violations when the numerical distance between the two amounts allocated is larger (e.g., a 7:1 violation), but not when it is smaller (e.g., a 3:1 violation). Regardless of which of these possibilities turns out to be correct (we return to them in the section “General Discussion”), the main thrust of the continuity hypothesis is that an expectation of fairness emerges very early in life, as part of the “first draft” of moral cognition (Graham et al., 2013).

Which of the two preceding hypotheses is more likely to be correct? Do infants acquire an expectation of fairness toward the end of the first year of life, as the shift hypothesis suggests, or is this expectation present beginning early in the first year but observable only under limited conditions, as the continuity hypothesis suggests? The present research sought to answer these questions.

### The Present Research

According to the continuity hypothesis, an expectation of fairness is present early in life but can initially be observed only under limited conditions. In particular, infants may initially be able to detect simple 2:0 violations, but not more challenging 3:1 violations. The present experiments tested three predictions from this hypothesis, using allocation-outcome tasks. A first prediction was that 9-month-olds would give evidence of sensitivity to fairness if presented with a 2:0 violation. Experiments 1 and 2 both tested this prediction, using slightly different procedures that made possible different control conditions. A second prediction, tested in Experiment 3, was that 9-month-olds would succeed in detecting a 2:0 but not a 3:1 violation. Finally, a third prediction was that infants younger than 9 months might also succeed in detecting a 2:0 violation. To evaluate this prediction, Experiment 4 tested 4-month-olds using a design similar to that of Experiment 1.

We reasoned that finding the predicted results in all four experiments (a) would confirm the positive findings of Meristo et al. (2016) with a 2:0 violation and extend them to younger infants, (b) would confirm the negative findings of Ziv and Sommerville (2017) with a 3:1 violation, and more generally (c) would provide evidence for the continuity hypothesis.

## Experiment 1

Experiment 1 examined whether 9-month-old infants would succeed in detecting a 2:0 fairness violation in an allocation-outcome task. Infants were assigned to an experimental or an inanimate-control condition and saw live events (adapted from Sloane et al., 2012) in which a female experimenter divided two cookies either fairly or unfairly between two puppets. Each infant sat on a parent’s lap facing a large puppet-stage apparatus; at the start of each trial, a supervisor lifted a curtain at the front of the apparatus. In each condition, infants received one familiarization trial and one test trial, and each trial had an initial phase and a final phase.

**FIGURE 1:**
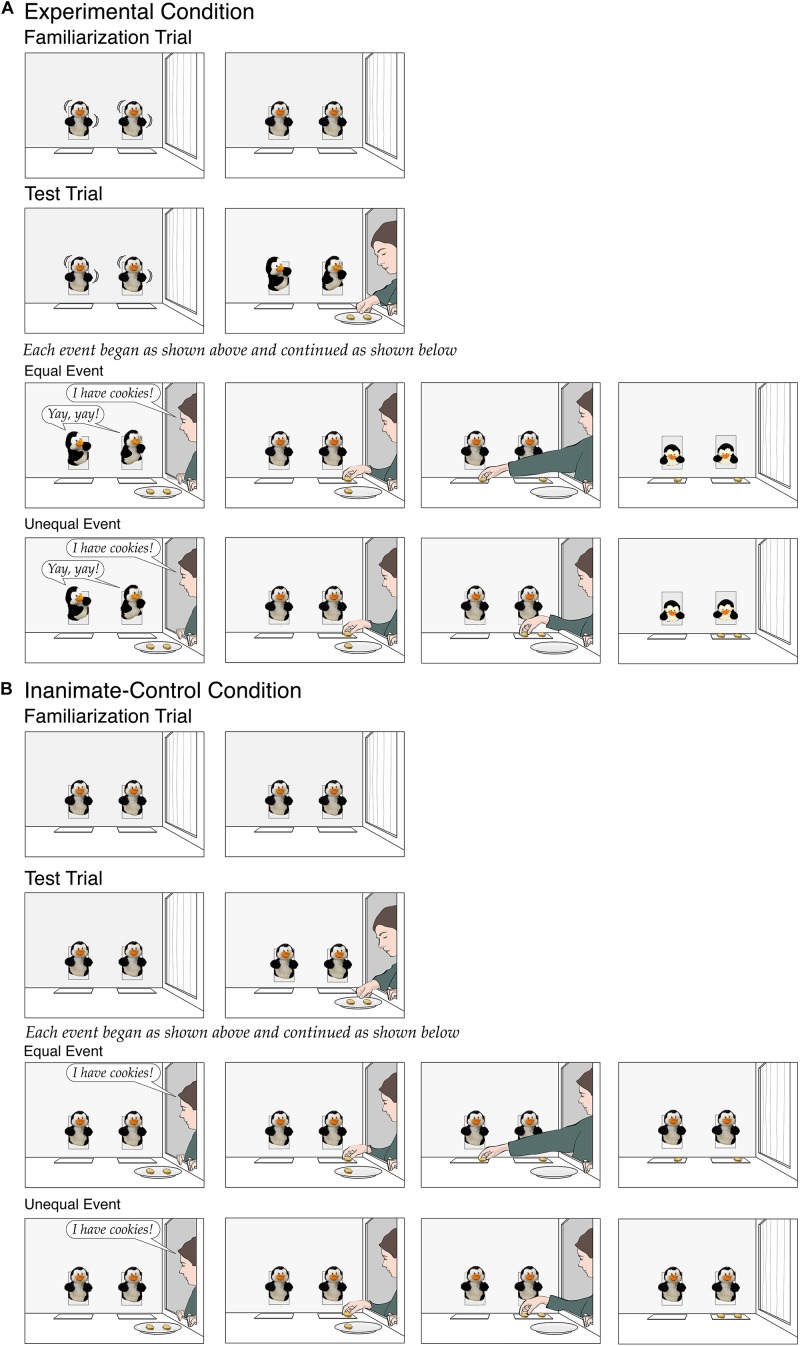
Schematic depiction of the events shown in the experimental condition **(A)** and the inanimate-control condition **(B)** of Experiment 1.

The familiarization trial served to introduce the puppets. At the start of the trial in the *experimental* condition (Figure 1A), two identical penguin puppets (operated by a hidden assistant) protruded from openings in the back wall of the apparatus; a small placemat lay in front of each puppet. During the initial (12-s) phase of the trial, the penguins “danced” by tilting from side to side every second. During the final phase, the penguins paused upright, and infants watched this paused scene until the trial ended (for criteria, see the section “Procedure”).

During the initial (26-s) phase of the test trial, the penguins danced until a female experimenter opened a curtained window in the right wall of the apparatus. The penguins turned toward her and watched as she brought in a plate with two identical cookies and placed it on the apparatus floor. The experimenter then announced, “I have cookies!,” and the penguins responded excitedly, “Yay, yay!” in two distinct female voices (the hidden assistant and the supervisor spoke in unison). Next, the experimenter placed a cookie on the placemat in front of one penguin (counterbalanced across infants); she then placed the other cookie in front of either the same penguin (*unequal* event) or the other penguin (*equal* event). Finally, the experimenter left with her empty plate, closing the curtain at her window, and the penguins looked down at their placemats and paused. During the final phase of the trial, infants watched this paused scene until the trial ended. We reasoned that if 9-month-olds expected the experimenter to divide the cookies fairly between the two puppets and could detect the 2:0 violation in the unequal event, then they should look significantly longer if shown that event as opposed to the equal event.

The *inanimate-control* condition (Figure 1B) served to rule out low-level interpretations of positive results in the experimental condition, such as a baseline preference for asymmetrical displays or for displays involving two cookies placed side by side. In previous experiments, researchers have consistently found that infants hold no expectation about how a distributor will divide windfall resources between two inanimate entities, suggesting that they appropriately restrict their expectation of fairness to animate entities (e.g., Sloane et al., 2012; Meristo et al., 2016; Ziv and Sommerville, 2017). In line with these findings, infants saw events identical to those in the experimental condition except that the penguins were inanimate: They did not move or talk and simply faced forward. Because the penguins gave no evidence of self-propulsion or agency (e.g., Setoh et al., 2013), we predicted that infants would view them as inanimate penguin-shaped toys, would hold no expectations about how the experimenter would divide the cookies between them, and hence would look about equally at the equal and unequal events.

### Materials and Methods

#### Sample-Size Considerations

In a recent report, Jin and Baillargeon (2017) examined sociomoral reasoning in infants using the violation-of-expectation method, a 2 × 2 between-subject design, and live events, as we did in the present research. The average Condition × Event effect size ($\eta_{p}^{2}$) in their experiments was 0.19. An *a priori* power analysis using G^∗^Power based on this value indicated that, with power set at 0.80 and alpha set at 0.05, the minimum number of participants required per cell (i.e., per combination of condition and event) was nine participants (Faul et al., 2007). In line with this analysis, our experiments used 10 participants per cell, for a total of 20 per condition and 40 per experiment.

Although this sample size is admittedly small and reflects the limitations of infant data collection in a small town, a number of considerations may help alleviate potential concerns. First, it should be noted that sample sizes of 8–12 infants per cell are common in violation-of-expectation tasks with between-subject designs, both in the area of sociomoral reasoning (e.g., Meristo and Surian, 2013; Jin and Baillargeon, 2017; Surian and Franchin, 2017; Bian et al., 2018; Surian et al., 2018; Wang and Henderson, 2018) and in other areas of infant cognition (e.g., Pitts et al., 2015; Kibbe and Leslie, 2016; Wellman et al., 2016; Scott, 2017; Stavans et al., 2018). Second, Experiments 2 and 3 provided conceptual replications of Experiment 1, again with 9-month-old infants. Third, following Experiment 3, we report an overall analysis of the pooled data from all three experiments (*n* = 120, with 30 infants per cell). Finally, following Experiment 4, which extended the results of Experiment 1 to 4-month-olds, we report a mini meta-analysis of the positive results from all four experiments. Thus, despite the relatively small number of infants per experiment, we believe that together, these multiple replications and overall analyses help provide a sound basis for our conclusions.

#### Participants

Participants were 40 healthy term 9-month-olds, 20 male (range = 8 months, 9 days to 10 months, 8 days, *M* = 9 months, 10 days). Another 10 infants were excluded, 7 because they looked for the maximum time allowed in the test trial,^2^ 1 because the infant was inattentive and looked away for 75% of the test trial, and 2 because their test looking times were over 3 standard deviations from the condition mean (both were in the experimental condition and saw the equal event). Half of the infants were randomly assigned to the experimental condition, and half to the inanimate-control condition; within each condition, half of the infants saw the equal event, and half saw the unequal event.

The infants’ names in all experiments were obtained from a university-maintained database of parents interested in participating in child-development research. Parents were offered either a small gift (e.g., a children’s book) or reimbursement for their travel expenses but were not otherwise compensated for their participation. Each infant’s parent gave written informed consent, and the protocol was approved by the Institutional Review Board at the University of Illinois at Urbana–Champaign.

#### Apparatus and Stimuli

The apparatus consisted of a brightly lit display booth (201 cm high × 102 cm wide × 58 cm deep) with a large opening (56 cm × 95 cm) in its front wall; between trials, the supervisor lowered a curtain in front of this opening. Inside the apparatus, the side walls were painted white, and the back wall and floor were covered with pastel adhesive paper.

The experimenter wore a green shirt, knelt at a window (51 cm × 38 cm) in the right wall of the apparatus, and slid a white curtain to open or close her window. Another curtain behind the experimenter hid the testing room. As the test trial unfolded, the experimenter looked naturally at the puppets and at the objects she acted on, but she never made eye contact with the infants.

The two puppets were identical penguins (about 22 cm × 12 cm × 9 cm at their largest points) made of black and white furry fabric; each penguin had a large head with an orange beak. The penguins protruded from openings (each 20 cm × 12.5 cm and filled with beige felt) located 20 cm apart in the back wall of the apparatus, 5 cm above the floor. In the experimental condition, an assistant sat behind the back wall and manipulated the penguins; in the inanimate-control condition, the penguins rested upright on hidden wooden posts. Centered beneath each penguin was a rectangular white placemat (0.5 cm × 20 cm × 13 cm). The cookies were plastic vanilla sandwich cookies (each about 1 cm × 3 cm × 7 cm), and they were introduced by the experimenter on a beige ceramic plate (2.5 cm × 20 cm in diameter).

To help the experimenter and the assistant adhere to the events’ scripts, a metronome beat softly once per second. During each testing session, one camera captured an image of the events, and another camera captured an image of the infant. The two images were combined, projected onto a computer screen located behind the apparatus, and monitored by the supervisor to confirm that the events followed the prescribed scripts. Recorded sessions were also checked off-line for experimenter accuracy.

#### Procedure

Each infant sat on a parent’s lap centered in front of the apparatus; parents were instructed to remain silent and to close their eyes during the test trial. Each infant’s looking behavior was monitored by two hidden observers who watched the infant through peepholes in cloth-covered frames on either side of the apparatus; the observers could not see the events from their viewpoints, and they did not know which test event was presented to the infant.^3^ Each observer held a game controller linked to a computer and pressed a button when the infant looked at the event. Looking times during the initial and final phases of each trial were computed separately, using the primary observer’s responses. Interobserver agreement during the final phase of each trial was measured as the proportion of 100-ms intervals in which the observers agreed about whether or not the infant was looking at the event; agreement was calculated for all 40 infants and averaged 93% per trial per infant.

Infants were highly attentive during the initial phases of the familiarization and test trials; across conditions, they looked, on average, for 93% of each initial phase. The final phase of each trial ended when infants (a) looked away for 2 consecutive seconds after having looked for at least 5 (familiarization) or 8 (test) cumulative seconds or (b) looked for a maximum of 45 cumulative seconds. A slightly longer minimum look was used in the test trial to give infants the opportunity to compare and evaluate the two puppets’ allocations before the trial could end.

Finally, preliminary analyses of the test data revealed no significant interaction of condition and event with infant’s sex or with which puppet received the first cookie, both *F*s(1,32) ≤ 1.41, *p* ≥ 0.244; the data were therefore collapsed across the latter two factors in subsequent analyses.

### Results and Discussion

Looking times during the final phase of the familiarization trial were subjected to an analysis of variance (ANOVA) with condition (experimental or inanimate-control) as a between-subject factor. This effect was not significant, *F*(1,38) = 0.22, *p* > 0.250, suggesting that infants in the experimental (*M* = 18.34, *SD* = 12.99) and inanimate-control (*M* = 16.38, *SD* = 13.27) conditions tended to look equally at the puppets (for data from all experiments, see Dataset in Supplementary Material).

**FIGURE 2:**
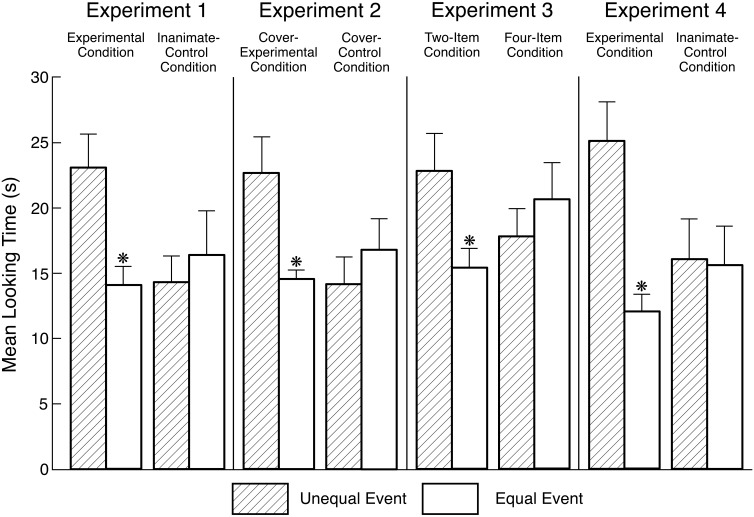
Mean looking times at the unequal and equal events during the final phase of the test trial in the various conditions of Experiments 1–4. The errors bars represent standard errors, and each asterisk denotes a significant difference between the two events within a condition (*p* < 0.05 or better).

Looking times during the final phase of the test trial (Figure 2) were subjected to an ANOVA with condition (experimental or inanimate-control) and event (unequal or equal) as between-subject factors. The only significant effect was the Condition × Event interaction, *F*(1,36) = 5.19, *p* = 0.029, $\eta_{p}^{2}$ = 0.13 (no such interaction was found in the familiarization trial, *F*(1,36) = 0.01, *p* > 0.250). Planned comparisons revealed that infants in the experimental condition looked significantly longer at the unequal (*M* = 23.04, *SD* = 8.11) than at the equal (*M* = 14.05, *SD* = 4.49) event, *F*(1,36) = 6.85, *p* = 0.013, Cohen’s *d* = 1.37, whereas infants in the inanimate-control condition looked about equally at the unequal (*M* = 14.28, *SD* = 6.36) and equal (*M* = 16.35, *SD* = 10.46) events, *F*(1,36) = 0.36, *p* > 0.250, *d* = 0.24. Non-parametric Wilcoxon rank-sum tests confirmed the results of the experimental (*Z* = 2.61, *p* = 0.009) and inanimate-control (*Z* = -0.26, *p* > 0.250) conditions.

Infants expected the experimenter to divide the two cookies equally between the two animated penguins, but they held no particular expectation about how the experimenter would divide the cookies between the two inanimate penguins. Together, these results provided evidence that 9-month-old infants already expect a distributor to divide two items equally between two similar recipients, thus supporting the continuity hypothesis.

## Experiment 2

Experiment 2 had two goals: One was to confirm the positive result of the experimental condition in Experiment 1, and the other was to address a possible alternative interpretation of this result. Specifically, infants might have looked longer at the unequal event not because they expected a distributor to divide windfall resources equally between similar individuals, but because they expected similar individuals to have similar numbers of objects (e.g., one cookie each; Welder and Graham, 2001). To rule out this alternative interpretation, infants in Experiment 2 were assigned to a cover-experimental or a cover-control condition. In the cover-experimental condition, the experimenter first removed covers placed over the penguins’ placemats and then proceeded to distribute the two cookies, as in Experiment 1. The cover-control condition (adapted from Sloane et al., 2012; see also Meristo et al., 2016) was identical except that the experimenter no longer brought in and distributed the two cookies: In each event, she simply removed the covers to reveal the cookies already resting on the penguins’ placemats. If infants merely expected similar puppets to have similar numbers of items, then infants in both the cover-experimental and cover-control conditions should look significantly longer at the unequal than at the equal event. However, if infants expected the experimenter to act fairly when she distributed the cookies to the puppets, but held no particular expectation about her actions when she simply revealed the cookies, then infants in the cover-experimental condition should look significantly longer at the unequal than at the equal event, whereas infants in the cover-control condition should look about equally at the two events.

**FIGURE 3:**
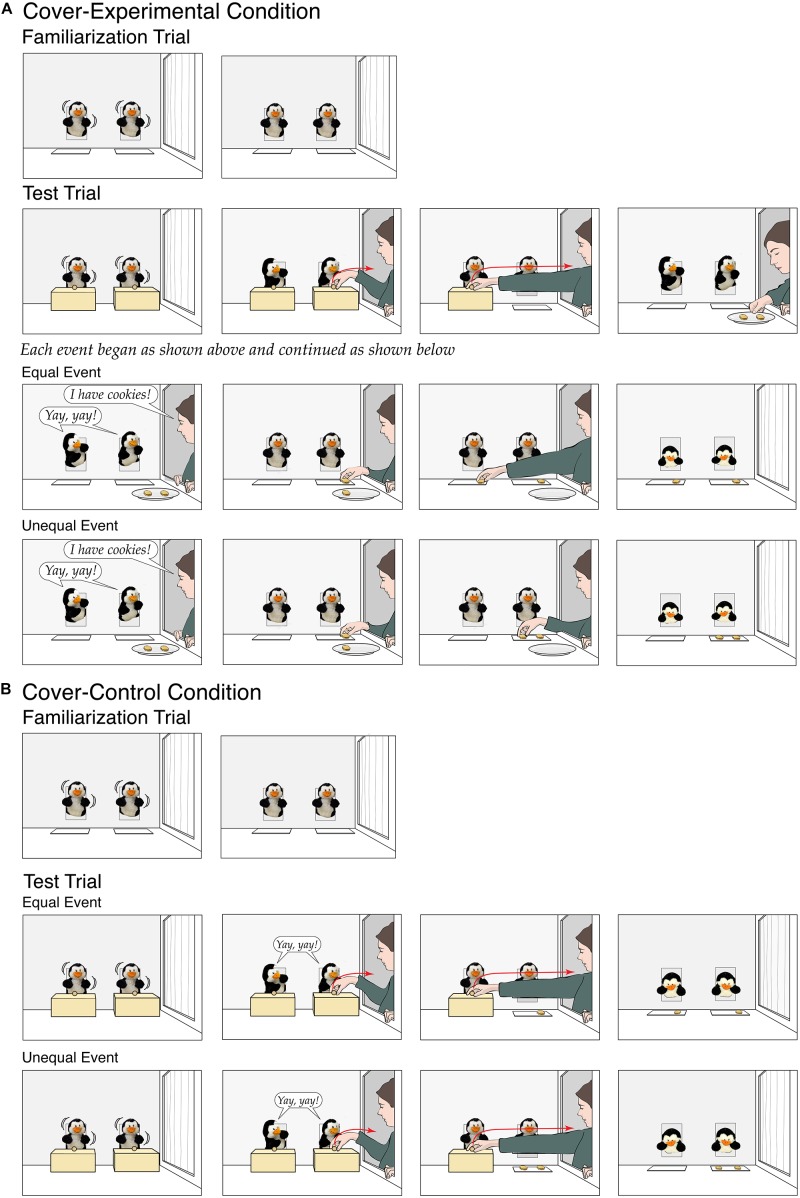
Schematic depiction of the events shown in the cover-experimental condition **(A)** and the cover-control condition **(B)** of Experiment 2.

Infants in the *cover-experimental* condition (Figure 3A) first received the same familiarization trial as in the experimental condition of Experiment 1, with the animated penguins dancing from side to side. Infants then received one test trial. At the start of the initial (42-s) phase, opaque rectangular covers rested in front of the penguins, over their empty placemats; the penguins (who were clearly visible above the covers) danced until the experimenter opened her window. The penguins then watched as the experimenter grasped one of the covers, lifted it, removed it from the apparatus through her window, and then repeated these actions with the other cover. Next, the experimenter brought in the plate with the two cookies, and the events proceeded exactly as in Experiment 1. Infants saw either the equal or the unequal event; for each event, which cover was removed first and which penguin received the first cookie were counterbalanced across infants.

The *cover-control* condition (Figure 3B) was identical with the following exceptions. At the start of the initial (26-s) phase of the test trial, the cookies were already on the penguins’ placemats, hidden under the covers. The experimenter removed the covers, one at a time, to reveal the cookies; in the unequal event, both cookies were in front of the same penguin; in the equal event, one cookie was in front of each penguin. The experimenter then left, and the penguins looked down at their placemats and paused. The experimenter did not speak in this condition, but the penguins did greet her (“Yay, yay!”) when she arrived. Which cover was removed first and which penguin had both cookies (unequal event only) were counterbalanced across infants.

### Materials and Methods

#### Participants

Participants were 40 healthy term 9-month-olds, 18 male (range = 8 months, 1 day to 10 months, 8 days, *M* = 9 months, 2 days). Another 12 infants were excluded, 6 because they looked for the maximum time allowed in the test trial,^2^ 4 because they were fussy (2), distracted (1), or subjected to parental interference (1), and 2 because their test looking times were over 3 standard deviations from the condition mean (one in each condition, and both saw the equal event). Half of the infants were randomly assigned to the cover-experimental condition, and half to the cover-control condition; within each condition, half of the infants saw the equal event, and half saw the unequal event.

#### Apparatus, Stimuli, and Procedure

The apparatus and stimuli were identical to those in Experiment 1, with the addition of two identical tan rectangular covers (each 10 cm × 22.5 cm × 15.5 cm, with a wooden knob at the top). The procedure was also identical to that in Experiment 1. Infants were highly attentive during the initial phases of the familiarization and test trials; across conditions, they looked, on average, for 97% of each initial phase. Interobserver agreement during the final phase of the test trial was calculated for all 40 infants and averaged 94% per trial per infant. Finally, preliminary analyses of the test data revealed no significant interaction of condition and event with infant’s sex or with which cover was removed first, both *F*s(1,32) ≤ 1.64, *p*s ≥ 0.209; the data were therefore collapsed across the latter two factors in subsequent analyses.

### Results and Discussion

Looking times during the final phase of the familiarization trial were analyzed by means of an ANOVA with condition (cover-experimental or cover-control) as a between-subject factor. This effect was not significant, *F*(1,38) = 0.30, *p* > 0.250, suggesting that infants in the cover-experimental (*M* = 20.52, *SD* = 12.70) and cover-control (*M* = 18.17, *SD* = 14.64) conditions tended to look equally at the puppets.

Looking times during the final phase of the test trial (Figure 2) were subjected to an ANOVA with condition (cover-experimental or cover-control) and test event (unequal or equal) as between-subjects factors. The only significant effect was the Condition × Event interaction, *F*(1,36) = 6.40, *p* = 0.016, $\eta_{p}^{2}$ = 0.15 (no such interaction was found in the familiarization trial, *F*(1,36) = 0.34, *p* > 0.250). Planned comparisons revealed that infants in the cover-experimental condition looked significantly longer at the unequal (*M* = 22.61, *SD* = 8.66) than at the equal (*M* = 14.56, *SD* = 1.98) event, *F*(1,36) = 7.26, *p* = 0.011, *d* = 1.28, whereas infants in the cover-control condition looked about equally at the unequal (*M* = 14.13, *SD* = 6.44) and equal (*M* = 16.77, *SD* = 7.63) events, *F*(1,36) = 0.78, *p* > 0.250, *d* = 0.37. Wilcoxon rank-sum tests confirmed the results of the cover-experimental (*Z* = 2.04, *p* = 0.041) and cover-control (*Z* = -0.87, *p* > 0.250) conditions.

When the experimenter brought in and distributed the two cookies, infants expected her to do so fairly, and they detected a violation when she instead gave both cookies to the same puppet. However, when the experimenter simply lifted covers to reveal the cookies already resting on the puppets’ placemats, infants held no particular expectation about how many cookies each puppet would have. Infants thus bring to bear considerations of fairness when resources are distributed between similar individuals, but not when resources already in individuals’ possession are revealed.

## Experiment 3

According to the continuity hypothesis, the discrepancy between the positive findings of Meristo et al. (2016) and the negative findings of Ziv and Sommerville (2017) was not due to the fact that the former tested 10-month-olds and the latter 9-month-olds; rather, it was due to the fact that the former used a simple 2:0 violation and the latter a more challenging 3:1 violation. Experiments 1 and 2 provided initial evidence for this hypothesis by showing that 9-month-olds could indeed detect a 2:0 fairness violation. Building on these results, Experiment 3 sought to confirm that 9-month-olds would detect a 2:0 violation (*two-item* condition), but not a 3:1 violation (*four-item* condition).

A secondary goal of Experiment 3 was to address a possible alternative interpretation of the positive results of Experiments 1 and 2: Infants might have looked longer at the unequal event not because they expected equal *distributions*, but because they expected equal *interactions*. In the cover-experimental condition of Experiment 2, for example, the experimenter first removed the cover from each puppet’s placemat – but then she gave both cookies to the same puppet, thereby excluding the disadvantaged puppet from these last interactions. Because infants and older children have been shown to be sensitive to exclusion cues (e.g., Tronick, 2007; Over and Carpenter, 2009; Abrams et al., 2011), these unequal interactions gave rise to the possibility that infants were showing sensitivity to exclusion, rather than to unfairness (e.g., DesChamps et al., 2016). Some evidence against this possibility came from a control condition by Meristo et al. (2016): Instead of distributing two strawberries either equally or unequally between two recipients, as in the experimental condition, the distributor performed the same actions without distributing strawberries (i.e., approached either each recipient in turn or the same recipient twice). Infants in this control condition looked about equally at the two events, suggesting that they held no expectation that the distributor would approach each recipient equally or include both recipients in its social exchanges.^4^ This negative result makes it unlikely that infants in the experimental condition of Meristo et al. (2016), or in the experimental conditions of Experiments 1 and 2, looked longer at the unequal event because they detected a violation when the distributor appeared to ignore the disadvantaged recipient when distributing items. Nevertheless, to provide additional evidence against this exclusion interpretation, in Experiment 3 we used a different distribution procedure, adapted from Schmidt and Sommerville (2011), which equated the distributor’s interactions with the two recipients, irrespective of whether distributions were equal or unequal. Specifically, rather than distributing each cookie one by one, the experimenter now divided the cookies between two placemats and then slid one placemat toward each puppet. With this mode of distribution, differences between the two conditions, or between the unequal and equal events within each condition, could not be attributed to differences in how many times the experimenter interacted with each puppet.

**FIGURE 4:**
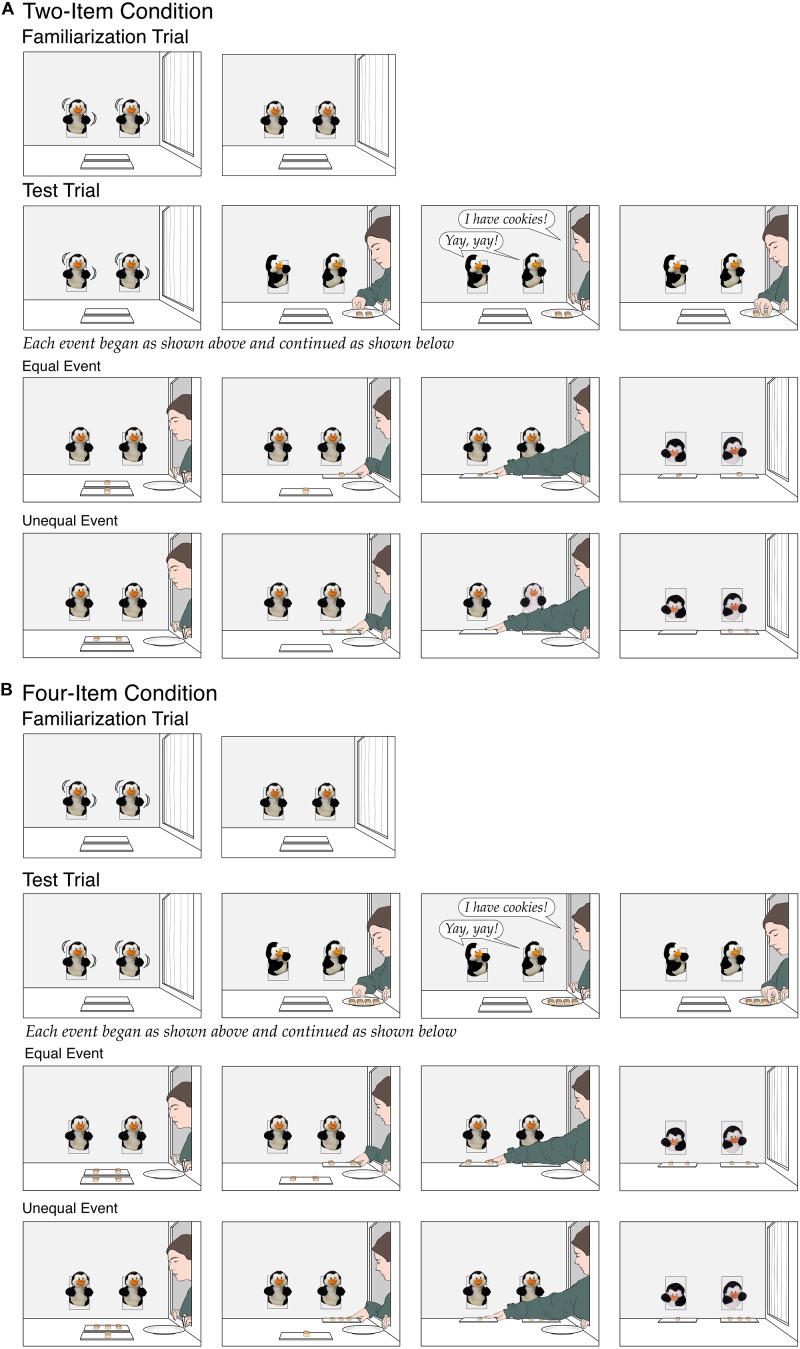
Schematic depiction of the events shown in the two-item condition **(A)** and the four-item condition **(B)** of Experiment 3.

Infants in both conditions first received the same familiarization trial as in the experimental condition of Experiment 1, with one exception: The placemats now rested back to back, 2.5 cm apart, at the front of the apparatus, centered between the two puppets in the back wall. Infants then received one test trial. At the start of the initial (33-s) phase in the *two-item* condition (Figure 4A), the penguins danced until the experimenter opened her window. As before, the experimenter brought in a plate with two cookies and announced, “I have cookies!,” to which the penguins responded, “Yay, yay!.” Next, the experimenter put one cookie on the back placemat and then one cookie on the front placemat (*equal* event), or she put both cookies, one at a time, on the back placemat (*unequal* event); the experimenter always started with the back placemat to make it easier for infants to see what was put on each placemat. The experimenter then paused briefly, to allow infants to compare the two placemats. Finally, the experimenter slid the back placemat toward one puppet and then the front placemat toward the other puppet. The experimenter then left, and the puppets looked down at their placemats and paused, as in Experiment 1. Each infant saw either the equal or the unequal event; in each event, which penguin received the back placemat was counterbalanced across infants. The *four-item* condition (Figure 4B) was identical with two exceptions. First, the experimenter brought in four cookies and either put two on each placemat (*equal* event) or put three on the back placemat and one on the front placemat (*unequal* event). Second, the initial phase of the test trial was extended from 33 to 39 s, as it took the experimenter slightly divide to divide four as opposed to two cookies.

Based on the positive results of Experiments 1 and 2, we predicted that the 9-month-olds in the two-item condition would again detect the fairness violation in the unequal event and hence would look significantly longer if shown that event as opposed to the equal event. In contrast, based on the negative results of Ziv and Sommerville (2017) with 9-month-olds, we predicted that infants in the four-item condition would be unable to detect the fairness violation they were shown and hence would tend to look equally at the unequal and equal events. Together, these results would provide strong evidence that young infants do possess an expectation of fairness but are initially very limited in the violations they can detect.

### Materials and Methods

#### Participants

Participants were 40 healthy term 9-month-olds, 17 male (range = 8 months, 1 day to 9 months, 29 days, *M* = 9 months, 1 day). Another 4 infants were excluded, 2 because they looked for the maximum time allowed in the test trial,^2^ 1 because the infant was distracted, and 1 because the infant’s test looking time was over 3 standard deviations from the condition mean (the infant was in the two-item condition and saw the equal event). Half of the infants were randomly assigned to the two-item condition, and half to the four-item condition; within each condition, half of the infants saw the equal event, and half saw the unequal event.

#### Apparatus, Stimuli, and Procedure

The apparatus and stimuli were identical to those in Experiment 1, with two exceptions: Four cookies were used in the four-item condition, and felt was attached to the undersides of the placemats so that they slid quietly on the apparatus floor. The procedure was also identical to that of Experiment 1. Infants were highly attentive during the initial phases of the familiarization and test trials; across conditions, they looked, on average, for 96% of each initial phase. Interobserver agreement during the final phase of each trial was calculated for all 40 infants and averaged 94% per trial per infant. Finally, preliminary analyses of the test data revealed no significant interaction of condition and event with infant’s sex or with which penguin received the back placemat, both *F*s(1,32) ≤ 1.61, *p*s ≥ 0.213; the data were therefore collapsed across these latter two factors in subsequent analyses.

### Results and Discussion

Looking times during the final phase of the familiarization trial were subjected to an ANOVA with condition (two- or four-item) as a between-subject factor. This effect was not significant, *F*(1,38) = 1.54, *p* = 0.222, suggesting that infants in the two-item (*M* = 15.80, *SD* = 8.58) and four-item (*M* = 20.22, *SD* = 13.40) conditions tended to look equally at the puppets.

Looking times during the final phase of the test trial (Figure 2) were subjected to an ANOVA with condition (two- or four-item) and test event (unequal or equal) as between-subject factors. The only significant effect was the Condition × Event interaction, *F*(1,36) = 4.59, *p* = 0.039, $\eta_{p}^{2}$ = 0.11 (no such interaction was found in the familiarization trial, *F*(1,36) = 1.55, *p* = 0.221). Planned comparisons revealed that infants in the two-item condition looked significantly longer at the unequal (*M* = 22.77, *SD* = 9.09) than at the equal (*M* = 15.37, *SD* = 4.79) event, *F*(1,36) = 4.83, *p* = 0.035, *d* = 1.01, whereas infants in the four-item condition looked about equally at the unequal (*M* = 17.76, *SD* = 6.76) and equal (*M* = 20.57, *SD* = 8.70) events, *F*(1,36) = 0.70, *p* > 0.250, *d* = 0.36. Wilcoxon rank-sum tests confirmed the results of the two-item (*Z* = 2.04, *p* = 0.041) and four-item (*Z* = -0.49, *p* > 0.250) conditions.

Consistent with the positive findings of Experiments 1 and 2, infants in the two-item condition detected a fairness violation when one puppet received a placemat with two cookies and the other puppet received a placemat with no cookies. Moreover, consistent with the negative findings of Ziv and Sommerville (2017), infants in the four-item condition failed to detect a violation when one puppet received a placemat with three cookies while the other puppet received a placemat with one cookie. Because the experimenter’s interactions with the puppets were identical in the two conditions (she simply slid one placemat toward each puppet), these diverging results most likely stemmed from the numbers of items involved in each violation: Infants were able to detect a 2:0 violation, but not a 3:1 violation. This last finding is particularly striking because the two placemats were initially positioned back-to-back at the front of the apparatus, making it easy for infants to determine via one-to-one correspondence that the back placemat had two more cookies than the front placemat. We return in the section “General Discussion” to possible reasons why infants still failed to detect this violation.

## Overall Analyses of Experiments 1–3

To test the robustness of our results, we conducted overall analyses of the test data from Experiments 1–3. In these analyses, we pooled the data from the experimental (Experiment 1), cover-experimental (Experiment 2), and two-item (Experiment 3) conditions into a *combined-experimental* condition (*N* = 60), and the data from the inanimate-control (Experiment 1), cover-control (Experiment 2), and four-item (Experiment 3) conditions into a *combined-control* condition (*N* = 60). As can be seen in Figure 2, infants in the first three conditions looked longer if shown the unequal as opposed to the equal event, suggesting that they detected a violation in the unequal event; in contrast, infants in the last three conditions tended to look equally at the two events, suggesting (at the very least) that they had no baseline preferences for asymmetrical displays or for displays depicting groups of two or three cookies.

Preliminary analyses of the test data in these combined-experimental and combined-control conditions revealed no significant interaction of condition and event with infants’ sex or with which puppet the experimenter approached first (in Experiment 1, when giving a cookie; in Experiment 2, when removing a cover; and in Experiment 3, when giving a placemat), both *Fs*(1,112) ≤ 0.98, *p*s ≥ 0.250; the data were therefore collapsed across the latter two factors in the following analyses.

We first conducted an ANOVA with condition (combined-experimental or combined-control) and event (equal or unequal) as between-subject factors. This analysis yielded a significant main effect of event, *F*(1,116) = 4.63, *p* = 0.034, and a significant Condition × Event interaction, *F*(1,116) = 16.52, *p* < 0.0001, $\eta_{p}^{2}$ = 0.12.^5^ As expected, planned comparisons revealed that infants in the combined-experimental condition looked significantly longer if shown the unequal (*M* = 22.81, *SD* = 8.33) as opposed to the equal (*M* = 14.66, *SD* = 3.86) event, *F*(1,116) = 19.33, *p* < 0.0001, *d* = 1.26, whereas infants in the combined-control condition looked about equally at the unequal (*M* = 15.39, *SD* = 6.52) and equal (*M* = 17.90, *SD* = 8.90) events, *F*(1,116) = 1.83, *p =* 0.179, *d* = -0.32. Wilcoxon rank-sum tests confirmed the results of the combined-experimental (*Z* = 3.96, *p* < 0.0001) and combined-control (*Z* = -0.91, *p* > 0.250) conditions.

Next, we focused on the combined-experimental condition only and examined the effects of two additional variables. The first was whether infants with and without older siblings differed in their ability to detect the violation in the unequal event. Recall that Ziv and Sommerville (2017) found that at 12–15 months, infants with siblings looked longer at a 3:1 than at a 2:2 outcome when both were displayed simultaneously, whereas infants without siblings looked about equally at the two outcomes. In the combined-experimental condition, 30 infants had one or more siblings (14 saw the unequal event and 16 saw the equal event), and 30 did not (the corresponding numbers were 16 and 14). Looking times were compared by means of an ANOVA with siblings (yes or no) and event (unequal or equal) as between-subject factors. The main effect of sibling was not significant, nor was the Sibling × Event interaction, both *F*s(1,56) ≤ 1.20, *p*s > 0.250. The only significant effect was the main effect of event, *F*(1,56) = 22.76, *p* < 0.0001, $\eta_{p}^{2}$ = 0.29. Planned comparisons indicated that infants with siblings, *F*(1,56) = 15.49, *p* = 0.0002, *d =* 1.35, and infants without siblings, *F*(1,56) = 7.90, *p* = 0.007, *d =* 1.10, both looked significantly longer if shown the unequal as opposed to the equal event (with siblings: unequal, *M* = 22.54, *SD* = 9.01, equal, *M* = 13.18, *SD* = 3.90; without siblings: unequal, *M* = 23.04, *SD* = 7.97, equal, *M* = 16.36, *SD* = 3.14). Wilcoxon rank-sum tests confirmed the positive results obtained with the infants with siblings (*Z* = 3.01, *p* = 0.003) and without siblings (*Z* = 2.33, *p* = 0.020). Infants in the combined-experimental condition were thus able to detect the simple 2:0 violation they were shown whether they had older siblings or not.

The second variable we explored was age. Since infants in the combined-experimental condition varied in age from 8 to 10 months, we divided them via a median split into a younger, 8-month-old group (*N* = 30, range = 8 months, 1 day to 9 months, 3 days, *M* = 8 months, 17 days) and an older, 9-month-old group (*N* = 30, range = 9 months, 7 days to 10 months, 8 days, *M* = 9 months, 18 days). In the younger group, 18 infants saw the unequal event, and 12 infants saw the equal event; in the older group, these numbers were reversed. Our main goal here was to establish whether the younger half of our sample was as likely as the older half to detect the 2:0 violation they were shown. This analysis was identical to that above except that sibling was replaced by age (8 or 9 months) as a between-subject factor. The main effect of age was not significant, nor was the Age × Event interaction, both *F*s(1,56) ≤ 0.10, *p*s > 0.250. Once again, only the main effect of event was significant, *F*(1,56) = 22.09, *p* < 0.0001, $\eta_{p}^{2}$ = 0.28. Planned comparisons indicated that 8-month-olds, *F*(1,56) = 12.62, *p* = 0.0008, *d =* 1.23, and 9-month-olds, *F*(1,56) = 9.58, *p* = 0.003, *d =* 1.35, both looked significantly longer if shown the unequal as opposed to the equal event (8 months: unequal, *M* = 22.98, *SD* = 9.36, equal, *M* = 14.24, *SD* = 3.76; 9 months: unequal, *M* = 22.55, *SD* = 6.87, equal, *M* = 14.94, *SD* = 4.00). Wilcoxon rank-sum tests confirmed the positive results obtained with the 8-month-olds (*Z* = 2.67, *p* = 0.008) and 9-month-olds (*Z* = 2.84, *p* = 0.005). Infants in the combined-experimental condition were thus able to detect the simple 2:0 violation they were shown whether they were 8 or 9 months of age.

## Experiment 4

**FIGURE 5:**
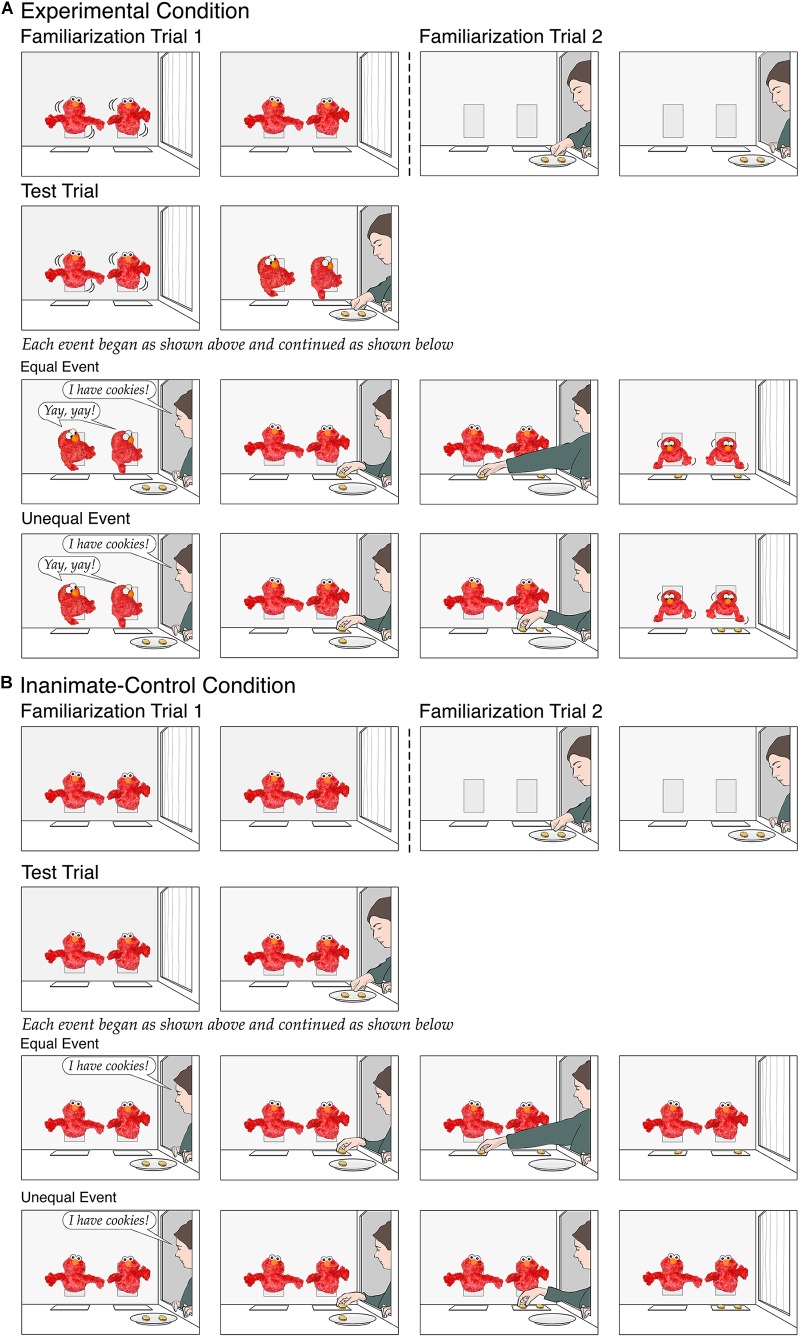
Schematic depiction of the events shown in the experimental condition **(A)** and the inanimate-control condition **(B)** of Experiment 4.

As predicted by the continuity hypothesis, the 8- and 9-month-olds in Experiments 1–3 could detect a simple 2:0 fairness violation but not a more challenging 3:1 fairness violation. In Experiment 4, we began to explore whether infants younger than 8 months might also be able to detect a 2:0 violation. Four-month-olds were tested using a design similar to that of Experiment 1; half of the infants were assigned to the experimental condition (Figure 5A), and half to the inanimate-control condition (Figure 5B).

To make our events more appropriate for these very young subjects, we introduced three modifications. First, we used Elmo puppets, whose bright red color and large eyes seemed likely to capture the attention of 4-month-olds. Second, we gave infants two familiarization trials. The first served to introduce the puppets and was similar to that in Experiment 1; in the experimental condition, the puppets danced from side to side, and in the inanimate-control condition, they remained stationary. The second trial served to introduce the experimenter. During the (6-s) initial phase, she opened her window, deposited her plate of cookies on the apparatus floor, and then paused for the final phase of the trial (the puppets were absent in this trial). Third, during the final phase of the test trial in the experimental condition, the puppets moved slightly from side to side while bent over their placemats (pilot data suggested that the sudden change from moving to still Elmos seemed to be upsetting for some infants; this was not an issue in the inanimate-control condition because the Elmos were inanimate throughout the trials).

We reasoned that if 4-month-olds already possess an expectation of fairness and can detect simple 2:0 fairness violations, then infants in the experimental condition should look significantly longer if shown the unequal as opposed to the equal event, whereas infants in the inanimate-control condition should look about equally at the two events, as in Experiment 1.

### Materials and Methods

#### Participants

Participants were 40 healthy term 4-month-olds, 20 male (range = 3 months, 21 days to 5 months, 18 days, *M* = 4 months, 21 days). Another 10 infants were excluded, 6 because they looked for the maximum time allowed in the test trial (4 were in the experimental condition, 2 were in the inanimate-control condition, and all saw the equal event), 2 because they were distracted or inattentive, and 2 because their test looking times were over 3 standard deviations from the condition mean (both were in the experimental condition and saw the equal event). Half of the infants were randomly assigned to the experimental condition and half to the inanimate-control condition; within each condition, half of the infants saw the equal event, and half saw the unequal event.

#### Apparatus, Stimuli, and Procedure

The apparatus and stimuli were identical to those in Experiment 1 except that the penguin puppets were replaced by two identical Elmo puppets (about 25 cm × 25 cm × 10 cm at the largest points). Each puppet was made of red furry fabric and had a large head, large black and white eyes, and an orange nose. The procedure was similar to that in Experiment 1, with two exceptions. First, as noted earlier, infants received two familiarization trials, one to introduce the puppets and then one to introduce the experimenter. Second, a slightly different look-away criterion was used to end the final phase of each trial. Each trial now ended when the infant looked away for 1 cumulative second, as opposed to 2 cumulative seconds. This adjustment was necessary because infants tended to look more continuously at the events, either because of their very young age, because they found the Elmo puppets highly eye-catching, or both.

Infants were highly attentive during the initial phases of the familiarization and test trials; across conditions, they looked, on average, for 87% of each initial phase. Interobserver agreement during the final phase of each trial was calculated for all 40 infants and averaged 92% per trial per infant. Finally, preliminary analyses of the test data revealed no significant interaction of condition and event with infant’s sex or with which puppet received the first cookie, both *F*s(1,32) ≤ 1.55, *p*s ≥ 0.222; the data were therefore collapsed across these latter two factors in subsequent analyses.

### Results and Discussion

Looking times during the final phase of the first familiarization trial (which introduced the puppets) were subjected to an ANOVA with condition (experimental or inanimate-control) as a between-subject factor. This effect was not significant, *F*(1,38) = 0.35, *p* > 0.250, suggesting that infants in the experimental (*M* = 22.17, *SD* = 15.67) and inanimate-control (*M* = 25.14, *SD* = 16.32) conditions tended to look equally at the puppets. Looking times during the second familiarization trial (which introduced the experimenter and her tray of cookies) were analyzed in the same manner. The main effect of condition was now significant, *F*(1,38) = 5.83, *p* = 0.021, indicating that infants in the inanimate-control condition (*M* = 21.28, *SD* = 17.75) looked significantly longer than those in the experimental condition (*M* = 11.29, *SD* = 5.21). It could be that infants in the inanimate-control condition found this trial more interesting because it involved an animate individual (recall that they had seen only the inanimate puppets in the previous trial), or it could be that infants in the experimental condition found this trial less interesting because the animated puppets introduced in the first trial were now absent. Either way, this finding did not affect our interpretation of the test trial and is not discussed further.

Looking times during the final phase of the test trial (Figure 2) were subjected to an ANOVA with condition (experimental or inanimate-control) and test event (unequal or equal) as between-subjects factors. The analysis yielded a significant main effect of event *F*(1,36) = 6.25, *p* = 0.017, as well as a significant Condition × Event interaction, *F*(1,36) = 5.45, *p* = 0.025, $\eta_{p}^{2}$ = 0.13 (no such interaction was found in either the first or the second familiarization trial, both *F*s(1,36) ≤ 0.16, *p*s ≥ 0.250). Planned comparisons revealed that infants in the experimental condition looked significantly longer at the unequal (*M* = 25.05, *SD* = 9.50) than at the equal (*M* = 12.02, *SD* = 4.19) event, *F*(1,36) = 11.69, *p* = 0.002, *d* = 1.77, whereas infants in the inanimate-control condition looked about equally at the unequal (*M* = 16.02, *SD* = 9.83) and equal (*M* = 15.57, *SD* = 9.28) events, *F*(1,36) = 0.01, *p* > 0.250, *d* = 0.04. Wilcoxon rank-sum tests confirmed the results of the experimental (*Z* = 3.14, *p* = 0.002) and inanimate-control (*Z* = 0.00, *p* > 0.250) conditions.

Next, we compared the test responses of the 4-month-olds in Experiment 4 to those of the 9-month-olds in Experiment 1, using an ANOVA similar to that above but with age as an added between-subject factor. The main effect of age was not significant, nor was the Age × Condition × Event interaction, both *F*s(1,72) ≤ 0.04, *p*s ≥ 0.250, suggesting that the two age groups responded similarly to the test events they were shown. Because slightly different procedures were used at the two ages, however, these negative results should be interpreted with caution.

Next, we compared the test responses of 4-month-olds in the experimental condition (*N* = 20) who had (9) or did not have (11) an older sibling. The data were subjected to an ANOVA with sibling (yes or no) and event (unequal or equal) as between-subject factors. Neither the main effect of sibling nor the Sibling × Event interaction were significant, both *F*s(1,16) ≤ 1.24, *p*s ≥ 0.250, suggesting that infants responded similarly whether or not they had an older sibling. Given the small numbers of participants involved, however, these results should again be interpreted with caution.

Like the 9-month-olds in Experiment 1, the 4-month-olds in Experiment 4 expected the experimenter to divide the two cookies equally between the two animated puppets, and this effect was eliminated when the puppets were inanimate. These results provide the first experimental demonstration that sensitivity to fairness can already be observed, at least under simple conditions, in the first half-year of life.

## Mini Meta-Analysis of Experiments 1–4

Finally, we conducted a mini meta-analysis of the experimental data in our four experiments (i.e., the data from the experimental, cover-experimental, two-item, and experimental conditions in Experiments 1–4, respectively). There was no evidence of heterogeneity of effects across experiments (Cochran’s *Q* tests, *p*s > 0.10), so a fixed-effects meta-analytic model was used. The meta-analytic estimates indicated that across experiments, infants looked significantly longer at the unequal than at the equal event, *d*+ = 1.34 [0.85, 1.82], *z* = 5.39, *p* < 0.001. The Rosenthal Fail-Safe tests suggested that 50 additional failed studies would be required to disprove this effect.

## General Discussion

The present experiments yielded five findings. First, at both 9 months (Experiments 1–3) and 4 months (Experiment 4), infants expected an experimenter to divide two cookies equally (1:1) between two similar animated puppets, and they detected a violation when she divided them unequally (2:0) instead. Second, infants demonstrated this expectation whether the experimenter gave the cookies one by one to the puppets (Experiments 1, 2, and 4) or first separated them onto two placemats and then gave each puppet a placemat (Experiment 3). Third, infants held no particular expectation about the experimenter’s actions when the puppets were inanimate (Experiments 1 and 4) or when the experimenter did not distribute the cookies but simply lifted covers to reveal them (Experiment 2). Fourth, at both 9 months (Experiments 1–3) and 4 months (Experiment 4), infants with or without older siblings were equally likely to detect the violation in the 2:0 outcome. Finally, when the number of cookies distributed was increased from 2 to 4, 9-month-olds failed to detect the violation in the 3:1 outcome (Experiment 3). Together, these results confirm and extend prior findings that 10- to 19-month-olds detected a violation when shown a 2:0 outcome (Sloane et al., 2012; Meristo et al., 2016; Bian et al., 2018), that 12-month-olds failed to detect a violation when shown a 3:1 outcome (Sommerville et al., 2013; Tatone and Csibra, 2018), and that 9- and 6-month-olds failed to look preferentially at a 3:1 over a 2:2 outcome when both were presented simultaneously (Ziv and Sommerville, 2017).

The evidence reported here that 9- and 4-month-olds consistently detected a 2:0 violation provides strong support for the suggestion, from researchers across the social sciences, that the “first draft” (Graham et al., 2013) of human moral cognition includes an abstract expectation of fairness (e.g., Shweder et al., 1997; Dawes et al., 2007; Jackendoff, 2007; Premack, 2007; Rai and Fiske, 2011; Sloane et al., 2012; Baumard et al., 2013; Graham et al., 2013; Baillargeon et al., 2015; Meristo et al., 2016; Bian et al., 2018; Buyukozer Dawkins et al., in press). Such an expectation might have gradually evolved in our species in part because it represents a cost-effective strategy for reducing the likelihood of future negative interactions (e.g., Baumard et al., 2013; Cosmides and Tooby, 2013; Bian et al., 2018). By adhering to fairness, a distributor avoids having to work out in each and every resource-allocation situation that a recipient is likely to be resentful if offered, for no obvious reason, less than an equal share of a windfall resource. Over evolutionary time, a genuine expectation of fairness could have emerged that bypassed these mentalizing efforts, reduced errors, and ultimately benefited the distributor as well as the recipients. From this perspective, it would make sense that infants’ concern for fairness would be highly abstract and would be brought to bear whenever they saw a distributor divide windfall resources between two similar recipients, be they two women, two speaking puppets, or two animated geometric figures with eyes (e.g., Schmidt and Sommerville, 2011; Sloane et al., 2012; Meristo et al., 2016).

At the same time, however, our findings and those of Sommerville and her colleagues (e.g., Sommerville et al., 2013; Ziv and Sommerville, 2017; see also Tatone and Csibra, 2018) make clear that there are sharp limits in young infants’ ability to detect fairness violations. In particular, 9-month-olds are able to detect 2:0 violations, but not 3:1 violations, even when the experimenter’s actions toward the recipients are identical (i.e., the experimenter slides a placemat toward each recipient). How can we explain these differential results? There are at least three possibilities.

First, it may be that young infants are able to process distributions that involve two items, but not distributions that involve four or more items, due to limitations in their information-processing capacity (e.g., Diamond, 2013). Thus, when there are two recipients and two items, infants can form an expectation, via simple one-to-one correspondence, about how many items each recipient will get (1:1), and they can compare this expectation to the observed distribution (1:1 or 2:0). When there are four or more items, however, this whole process becomes overwhelming, leading to equal looking times at equal and unequal distributions.

Second, it may be that young infants are able to detect qualitative violations, in which one recipient gets something and the other gets nothing (e.g., a 2:0 or a 4:0 violation), but not quantitative violations, in which both recipients get something but in differing amounts (e.g., a 3:1 or a 7:1 violation). For example, infants’ representations of resource-allocation events could at first be very sparse: They might simply represent *whether* each recipient gets any items, rather than *how many* items each recipient gets. Such meager representations, when interpreted in light of infants’ expectation of fairness, would enable them to detect qualitative violations (i.e., something vs. nothing), but not quantitative violations (something vs. something). As a point of comparison, the physical-reasoning literature presents many examples of event representations that are initially very sparse and become progressively richer as infants identify relevant features that help better predict outcomes (for reviews, see Baillargeon et al., 2009; Stavans et al., 2018).

Finally, it may be that young infants can detect quantitative violations, but only when the two amounts allocated are markedly different. In this view, infants would succeed when the numerical distance between the two amounts is larger (e.g., a 7:1 violation), but not when it is smaller (e.g., a 3:1 violation), most likely due to limitations in their numerical cognition. With experience, infants would come to more precisely represent the amounts allocated to the two recipients and hence would begin to detect a deviation from fairness even in a 3:1 violation.

Which (if any) of the preceding possibilities might be correct? Can prior findings on when infants first begin to detect 3:1 violations help us distinguish between them? It is not clear that this is the case. In particular, consider the finding that 12–15-month-olds with older siblings looked significantly longer at a 3:1 than at a 2:2 outcome when the two were presented simultaneously (Ziv and Sommerville, 2017). These findings could be taken to suggest that, due to greater opportunities to represent and compare allocations in everyday life, infants with older siblings (a) are better at processing distributions with more than two items, (b) are faster at learning to attend not only to whether recipients get something but also to how many items they get, and/or (c) are more adept at precisely representing and comparing how many items recipients get. Future research can bear on these issues by examining whether young infants would succeed in detecting more extreme quantitative violations, such as a 5:1 or 7:1 violation. If yes, such results would tend to cast doubt on the first and second possibilities listed above and to support the third possibility instead. Such results would also dovetail well with recent findings that preschoolers sometimes perform poorly in first- and third-party fairness tasks due to cognitive limitations in their ability to encode and remember exact numerical information (e.g., Chernyak et al., 2016, 2019; Chernyak and Blake, 2017).

### Prior Findings With Young Infants

As noted above, the positive results obtained with 9- and 4-month-olds in the present allocation-outcome tasks confirm and extend those previously obtained with 10-month-olds (Meristo et al., 2016). The present results also fit well with the finding that 10-month-olds (a) looked significantly longer when an informed bystander rewarded an unfair as opposed to a fair distributor, but (b) looked about equally when an uninformed bystander (whose view was blocked during the distributors’ actions) rewarded either distributor (Meristo and Surian, 2013). At the same time, however, our results and those just cited are inconsistent with a few other findings with young infants mentioned in the section “Introduction.”

One such finding was that after watching a fair and an unfair distributor divide two items between two recipients, 10-month-olds did not show a preference for the fair distributor (Geraci and Surian, 2011). Given the extensive evidence that infants in the first year of life prefer individuals who act positively over individuals who act negatively (e.g., Hamlin et al., 2007; Hamlin and Wynn, 2011; Hamlin, 2013a), it is unlikely that infants failed to prefer the fair distributor because they were too young to show such affiliative preferences. Rather, it is more likely that details about the task made it too difficult for young infants to process. In particular, the task involved five kinds of animal characters. To start, a bear or a lion (the distributor) stood alone at the center of the computer monitor, near two allocation items. Next, a chicken (an observer) entered the scene, brought the items closer to the distributor, and then rested at the bottom of the monitor. Next, a donkey and a cow (the recipients) entered one at a time and took positions in the top two corners of the monitor. Finally, the distributor divided the two items between the recipients, either equally (e.g., the bear) or unequally (e.g., the lion). Given this fairly complex cast of characters, infants might simply have had difficulty remembering who played what role in the events, due to their limited information-processing capacity. Future research can examine whether young infants might be more likely to succeed if shown simpler events involving a fair distributor (e.g., a bear), an unfair distributor (e.g., a lion), and two similar recipients (e.g., two donkeys). Given the present results, we would predict that even young infants would prefer the fair over the unfair distributor.

The other inconsistent findings were that after watching a fair and an unfair distributor divide two items between two similar recipients, 10-month-olds looked significantly longer when a newcomer *either* rewarded or punished the unfair as opposed to the fair distributor (Meristo and Surian, 2013, 2014). One possible explanation for these results is that because infants could form no particular expectations about the newcomer’s actions (recall that the newcomer was entirely absent during the distributor’s actions), their responses were guided primarily by a vigilance or negativity bias (e.g., Kinzler and Shutts, 2008; Vaish et al., 2008; Baltazar et al., 2012; DesChamps et al., 2016). Specifically, infants looked longer whenever the newcomer approached the unfair distributor, as though they were interested in monitoring and learning more about that distributor.

## Conclusion

In four experiments using allocation-outcome tasks, 9- and 4-month-olds detected a violation when shown an unfair 2:0 outcome. In contrast, 9-month-olds failed to detect a violation when shown an unfair 3:1 outcome. Together, these results support claims that an abstract expectation of fairness is a part of the basic structure of human moral cognition, but they also point to sharp limitations in young infants’ ability to detect deviations from fairness. The present results thus pave the way for future investigations of how numerical accuracy and other factors may contribute to the development of early expectations about fairness in infancy and beyond.

## Author Contributions

MBD, SS, and RB designed the experiments. MBD and SS performed the experiments. MBD analyzed the data. MBD and RB wrote the manuscript, with edits by SS.

## Conflict of Interest Statement

The authors state that they have no affiliations or involvement in any organization or entity with any financial or non-financial interest in the subject matter or materials discussed in this manuscript.

## Footnotes

1 Although Meristo and Surian (2013) described their task as a reward task, it could also be construed as an affiliative-preference task: Perhaps infants simply expected the bystander to prefer and approach the fair over the unfair distributor. In this view, the same results would have been obtained had the bystander simply approached each distributor, without giving them a strawberry (for evidence that young infants both form affiliative preferences and expect others to share these preferences, see Hamlin et al., 2007).

2 Across Experiments 1–3, 15 of the total 146 9-month-olds tested (10%) were excluded because they looked for the maximum amount allowed in the test trial (i.e., “reached ceiling”). The distribution of these 15 infants, in terms of the condition they were assigned to (and the test event they saw), was: Experiment 1, 4 experimental (1 unequal, 3 equal) and 3 inanimate-control (1 unequal, 2 equal); Experiment 2, 3 experimental (equal) and 3 cover-control (1 unequal, 2 equal); and Experiment 3, 1 experimental (equal) and 1 control (unequal). Ceiling infants are typically eliminated on the assumption that they needed additional familiarization to process the events they were shown (for other reports with eliminated ceiling babies, see e.g., Scott et al., 2015; Baillargeon and DeJong, 2017; Jin et al., 2018; Margoni et al., 2018). In line with this assumption, the ceiling infants in Experiments 1–3 looked significantly longer during the familiarization trial (*n* = 15, *M* = 33.77, *SD* = 20.62) than did the infants included in the experiments (*n* = 120, *M* = 18.24, *SD* = 12.59), *F*(1,133) = 17.24, *p* < 0.0001. Of course, these ceiling infants might have performed better had they been provided with more or more varied familiarization trials, for a better introduction to the task. However, because increasing the number of familiarization trials also increases the risk of inadvertently inducing subtle novelty or familiarity preferences that can then affect test responses (e.g., Wang et al., 2004), researchers often err in the direction of using as few familiarization trials as possible, and we followed this practice here.

3 At the end of the test trial in each experiment, the primary observer was asked to guess whether the infant had seen an unequal or an equal event during the trial. Across Experiments 1–4, the primary observer guessed correctly for 20/40, 20/38 (the observer failed to make a guess for two infants), 20/40, and 20/40 infants, respectively, all *p*s > 0.250 (cumulative binomial probability).

4 In their experimental condition, Meristo et al. (2016) hid the distributor’s actions by placing a large occluder at the center of the scene; at the end of each event, this occluder was removed to reveal that each potential recipient had received one strawberry (equal event) or that one recipient had received both strawberries (unequal event). This condition might also be taken to provide evidence against the exclusion interpretation, because infants did not witness the interactions between the distributor and recipients. However, because infants could easily infer what interactions had taken place behind the occluder, these results do not conclusively rule out the exclusion interpretation.

5 This interaction remained significant when the five outliers from Experiments 1–3 were included in the analyses (*n* = 125), *F*(1,121) = 10.42, *p* = 0.002, $\eta_{p}^{2}$ = 0.08.

## Supplementary Material

TABLE S1 Participant information and looking-time data for Experiments 1 through 4.
